# Risk Inequality and the Food-Energy-Water (FEW) Nexus: A Study of 43 City Adaptation Plans

**DOI:** 10.3389/fsoc.2019.00031

**Published:** 2019-04-18

**Authors:** Patricia Romero-Lankao, Daniel Gnatz

**Affiliations:** ^1^National Center for Atmospheric Research, Boulder, CO, United States; ^2^National Renewable Energy Laboratory, Golden, CO, United States; ^3^Mansueto Institute for Urban Innovation, University of Chicago, Chicago, IL, United States; ^4^Institute for Sustainable Urban Transformation, Boulder, CO, United States

**Keywords:** inequality, FEW nexus, urban adaptation, discourse analysis, meta-analysis, urban climate governance

## Abstract

Goals aimed at adapting to climate change in sustainable and just ways are embedded in global agreements such as the Sustainable Development Goals and the New Urban Agenda. However, largely unexamined, are the ways that narrative understandings conveyed in adaptation plans consider and attempt to address inequality in climate risk to urban populations and FEW-systems. In this paper, we examine whether and how adaptation plans from C40 member cities address inequality in risk, by planning actions to reduce hazard exposure or tackling the drivers of social vulnerability. C40 is a network of 94 of the world's cities fostering policies to address climate change. We apply a mixed methods approach, including a discourse analysis and meta-analysis of adaptation plans. The discourse analysis helps to unpack framings of urban equity issues as they relate to policy actions, and the meta-analysis seeks to quantitatively investigate patterns of framing and policy across adaptation plans. Our findings suggest that FEW-nexus thinking is not yet embedded in narrative understandings of risk and planned adaptation actions, within the adaptation plans we studied. In the city adaptation plans we analyzed, we found multiple frames coexisting behind the broader adaptation visions (e.g., risk and resilience). Rather than converging, issues, and principles such as those of equality, coexist with economic issues in an imbalance of incongruent political movements and priorities. Techno-infrastructural and economic investments and concerns tend to take precedence over concerns and interests for inequality in climate risks. We discuss some of the institutional factors explaining this. Knowledge integration, for instance, is constrained by the existence of a plurality of sectors, levels of government, power, values, and ways of understanding and managing climate risk. We also suggest that the relatively low importance of equality considerations in the adaptation plans will likely limit the capacity of cities to support broader goals such as those of the New Urban Agenda and the Sustainable Development Goals.

## Introduction

Goals aimed at adapting to climate change in sustainable and just ways are embedded in global agreements such as the Paris Agreement, Sustainable Development Goals and the New Urban Agenda. These agreements seek to move environmental and climate concerns into the urban policy action arena by developing strategies for risk management. Ideally, these strategies would be supported by the three pillars of sustainability (economy, equality, and environment), while increasing cities' resilience to chronic and acute physical, social, and economic stressors and hazards (Zeemering, 2009; Campbell, 2013; Romero-Lankao et al., 2016a; Simon et al., 2016). However, in practice, tradeoffs are often present that shrink the size one pillar and augment another.

In the last decade, scholars and decisionmakers have shown increased interest in the mechanisms by which urbanization and climate change are coevolving to compound the unequal risk of floods, wildfires, and other hazards to urban populations and their supporting food, energy, and water (FEW) systems. However, actions to improve equality on the ground have been less evident (Revi et al., 2014; Romero-Lankao et al., 2017c). Incorporation of equality into urban adaptation plans is important because the most vulnerable communities within cities, most often are more exposed, have lower socio-economic status, make lower contribution to GHG emissions, and have lower levels of access to FEW systems, and livelihood options to mitigate risk and adapt (Boone, 2010; Hughes, 2013; Agyeman et al., 2016; Romero-Lankao et al., 2016a; Shi et al., 2016; Reckien and Lwasa, 2017).

**Figure 1:**
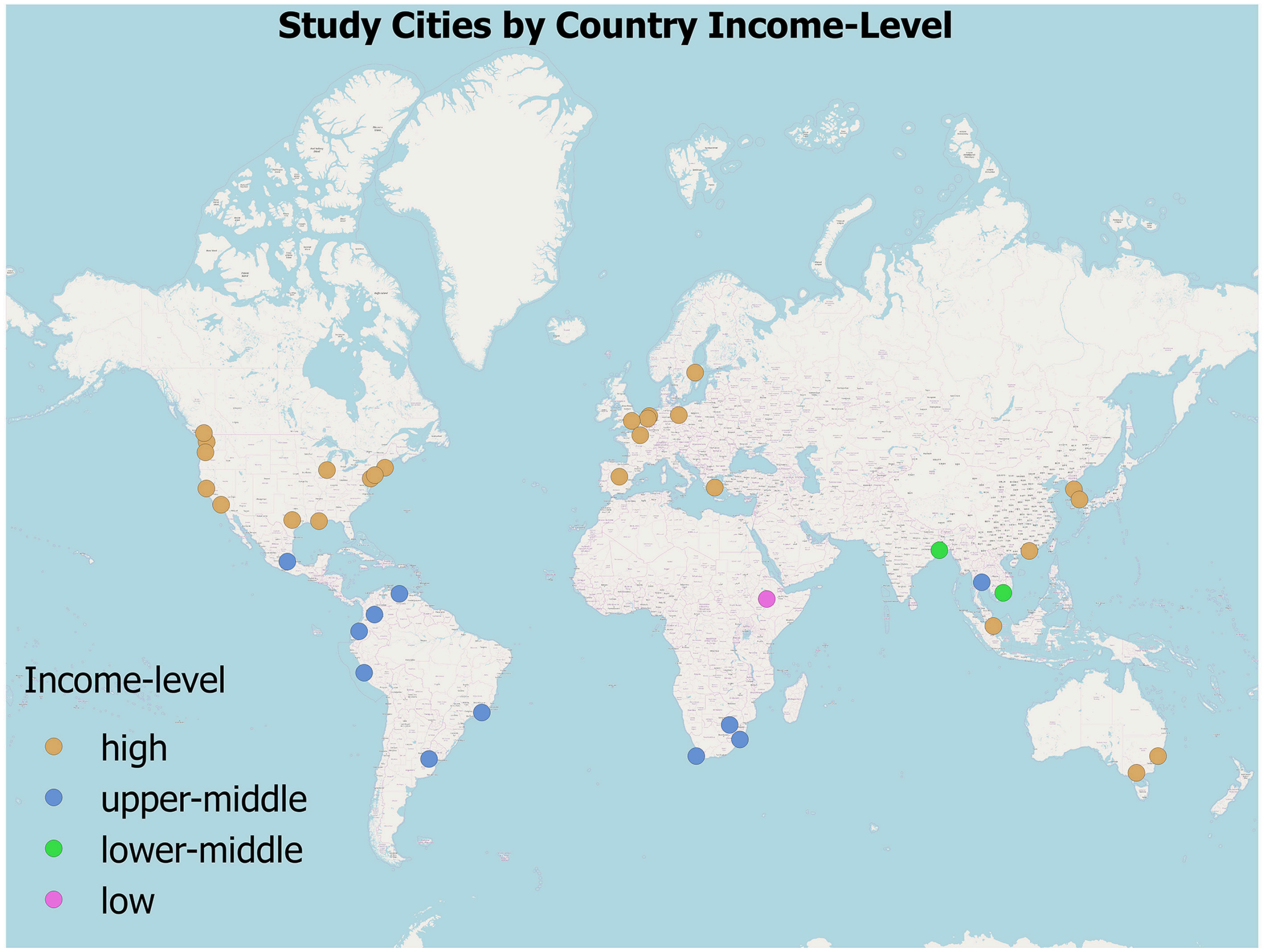
Cities covered in the analysis of adaptation plans. Based on World Bank income category as of 1 July 2015, at the country-level. Low-income economies are those with a GNI per capita, of $1,045 or less in 2014; middle-income economies are those with a GNI per capita of more than $1,045 but < $12,736; high-income economies are those with a GNI per capita of $12,736 or more. Lower-middle-income and upper-middle-income economies are separated at a GNI per capita of $4,125.

It is widely accepted, in the literature of social vulnerably, that social inequality shapes differences in climate risk and vulnerability and in capacity to mitigate and adapt to these hazards (Ribot, 2010; Romero-Lankao et al., 2016a). However, largely unexamined, are the ways in which different narrative understandings relate to suggested actions in existing adaptation plans. In this paper, we examine whether and how adaptation plans from 43 C40 cities address inequality in risk, by planning ways to reduce inequality in hazard exposure or tackling the drivers of social vulnerability (Reckien and Lwasa, 2017). We apply a mixed methods approach, including a discourse analysis and meta-analysis of adaptation plans for 43 C40 cities (Figure 1 and Supplemental Table 1A). In this approach, the discourse analysis helps unpack framings of urban equality issues as they relate to policy actions, and the meta-analysis seeks to quantitatively investigate patterns of framing and policy across adaptation plans.

## Tracing Existing Scholarship

Three areas of scholarship, relevant to this paper, include urban adaptation, and governance, inequality in climate risk, and the food, energy, and water (FEW) nexus (Leck et al., 2015; Araos et al., 2016; Shi et al., 2016; Romero-Lankao et al., 2017c; Wiegleb and Bruns, 2018; Heikkinen et al., 2019). We use findings in these areas as a basis to suggest a conceptual framework (section Conceptual Framework), which will be used to map attention given, in urban adaptation plans, to FEW interactions with inequality, and thereby gain knowledge of how far these considerations have penetrated urban adaptation planning.

### Urban Adaptation and Climate Governance

Having proven to be important agents of change globally, cities, and transnational networks occupy a central role in the global governance of climate change because of many reasons (Bulkeley and Betsill, 2013; Romero-Lankao et al., 2018). There is a wide acknowledgment among scholars of the incapacity of national actors alone to produce policy actions that can address the complex dynamics of climatic risk (Gordon and Johnson, 2017). Attention has shifted to the array of governance initiatives undertaken outside of interstate climate negotiations and policies. These initiatives, taken by state, municipal, market, and civil society actors operating at multiple local to global levels, are seen as key to creating the kinds of innovations necessary to address environmental change and climate risk (Acuto, 2013; Shi et al., 2015; Gordon and Johnson, 2017). In recent years, in what has been termed the second wave of urban climate governance (Bulkeley, 2010), cities have moved beyond symbolic commitment to climate change action, to its integration into their planning and development policies (Aylett, 2014). For many cities, part of this movement has included participation in local and city-networks such as ICLEI, the World Association of Major Metropolises (Metropolis) and the C40 Cities Climate Leadership Group (C40) (Bouteligier, 2013; Gordon and Johnson, 2017).

C40 is a network of 94 of the world's cities concentrating more than 650 million people and one quarter of the global economy. This peer network of cities seeks to address climate change through the design and implementation of policies seeking to mitigate greenhouse gas (GHG) emissions and climate risks (https://www.c40.org, February 2*8t*h,*2019*). A body of literature has examined different aspects of the C40 global and city governance influence. For instance, some portray the C40 as an orchestrator of global urban climate governance steering member cities toward particular climate actions (Gordon and Johnson, 2017), or creating new inequalities and sometimes even intensifying existing ones (Bouteligier, 2013). Others analyze whether the kind of change the network promotes is incremental, reformistic, or transformational (Heikkinen et al., 2019).

In this study, we start from the assumption that member city agendas may differ from that of the C40 network (Heikkinen et al., 2019), and examine how, in their adaptation plans, city officials understand and manage inequality in climate risk to urban populations and FEW-systems.

### Risk and the FEW-nexus

Studies on FEW nexus have grown recently (Endo et al., 2015). As it pertains to human food, energy, and water systems, the term nexus refers to the relationships, as defined by linkages and interdependencies, between two or more FEW resources and systems, including trade-offs and feedbacks between them (Leck et al., 2015; Romero-Lankao et al., 2017c). FEW-nexus scholarship has grown in recent years, but differences in motivation, purpose, and scope pervade the field (Stringer et al., 2018). A FEW-nexus approach can be used to analytically examine links and interdependencies between FEW-systems, but it also functions as a *boundary object* that engages decision makers and academics across a science-policy interface aimed at understanding and managing FEW-system links and interdependencies (Wiegleb and Bruns, 2018). In governance, its concepts are sometimes used to achieve integrated management across FEW sectors and jurisdictions (Bizikova et al., 2013).

Here we will examine how linkages and interdependencies between FEW-systems are acknowledged and prioritized at the city level and whether integrated FEW-management is a goal of adaptation plans. Or if, as suggested by existing scholarship, bringing together diverse policy domains creates its own set of challenges. The most important is given by the difficulties involved in moving decision makers beyond their accustomed ways of understandings and action precisely because this involves a collective engagement of disparate sectors, ways of knowing, levels of government, power, and values (Romero-Lankao et al., 2017c).

**Figure 2:**
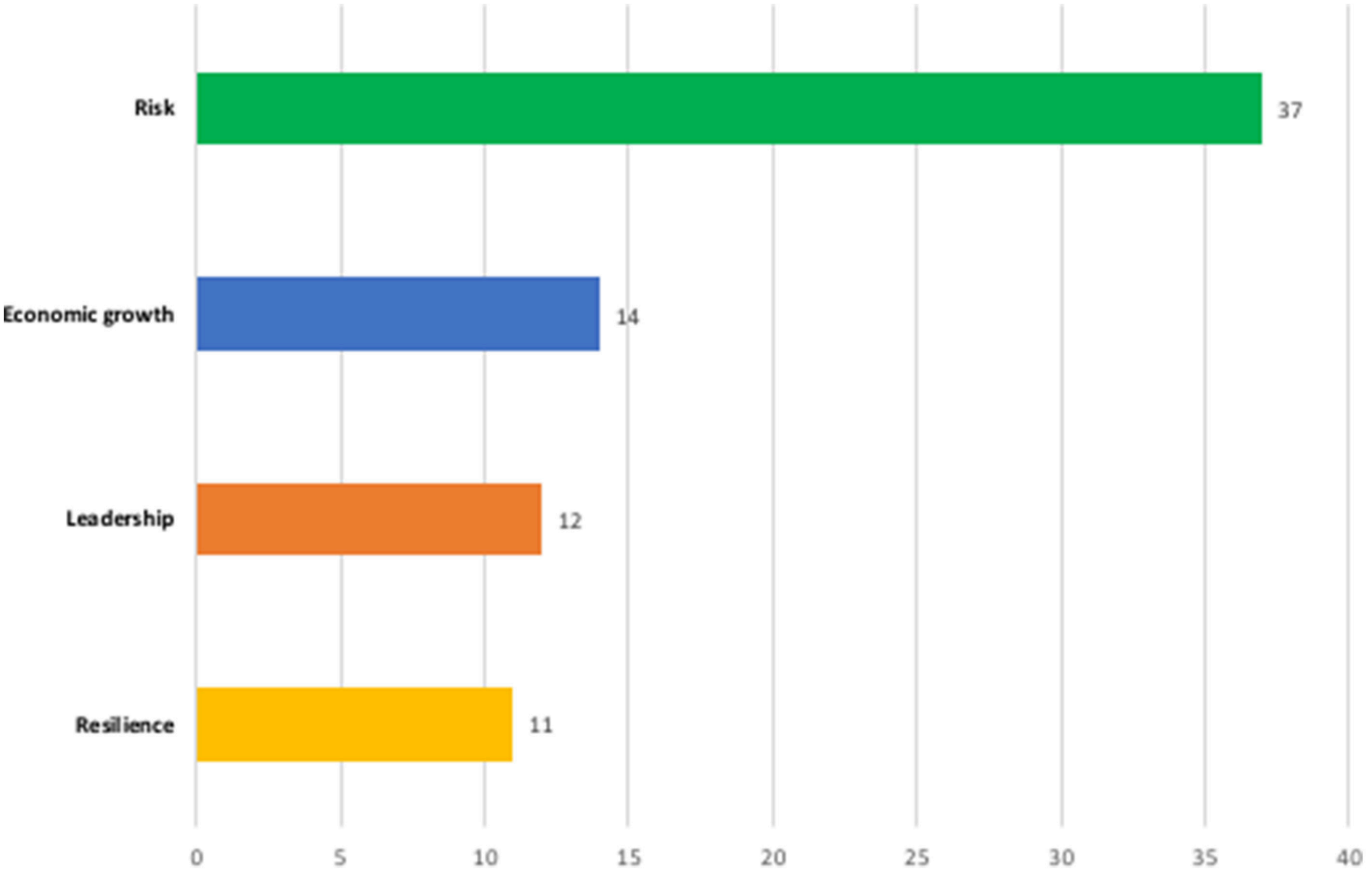
Framing the adaptation vision. After reading and summarizing each adaptation plan, four notions capturing cities' broader frame or vision were identified. See Supplemental Table 1B.

FEW-nexus studies tend to be motivated either by the scarcity of FEW resources or by threats to FEW-resource security due to development and environmental pressures (Galaitsi et al., 2018). We will focus on the latter, which tends to be framed using either a security or a risk approach (Corry, 2012). In the security approach, the focus is on an existing threat such as an ongoing drought or disruption of energy or food supplies (Comfort, 2005). In the risk approach, however, the emphasis is on how human development and environmental dynamics are interplaying (or might interplay) to create the potential for harmful events (Trombetta, 2008). While security thinking leads decision making to look for the current, direct causes of harm to urban populations and FEW-systems, risk analysis analyzes the potential causes of harm, current or future. We use a risk approach here, because it fits better with both climate change scholarship, ours included, and the framing used in 87% of the adaptation plans (Field et al., 2014; Romero-Lankao et al., 2017a) (Figure 2).

Within our sample, we look at how adaptation plans address inequality in risk. Following the IPCC, we define risk as the potential for adverse effects on lives, livelihoods, health, and assets (Field et al., 2014). Risk may spring from exposure to floods, sea level rise, and other threats and vulnerability of people and the FEW-systems that support them. Such vulnerability, or the propensity to be negatively affected by events or impacts, results from the multiscale interplay of factors in five domains: ***S***ocio-demographic, **E**conomic, **T**echno-infrastructural, ***E***nvironmental, and **G**overnance (SET*E*G), which have been used by Arup and by us in prior work (Arup, 2014; Romero-Lankao and Gnatz, 2016). While people can be susceptible to hazards, they also have capacity and agency to modify their circumstances and behavior to mitigate risks or adapt. Capacity is the unequally distributed pool of resources, assets, and options governmental, private, and non-governmental actors can draw on to mitigate and adapt to risks, while pursuing their development goals and values (Vincent, 2007).

To understand how policymakers are prioritizing these issues, we examine how in their adaptation plans, city officials attribute climate risk to a series of locational and SET*E*G factors, and what policy actions they suggest to manage these (section Study Design).

### Urban Adaptation, Inequality, and Equality

For centuries, the notions of inequality, equality, and justice have been the subject of compelling philosophical, conceptual, and ethical debates, with persistent disagreements in definition, scope and policy implications whose discussion is beyond the scope of this paper (Ikeme, 2003; Agyeman et al., 2016).

The concepts of fairness and justice can be related to discussions of the differences in definitions of equal and equitable. The word justice comes from the Latin *jus*, meaning right or law, and refers to either an actual or ideal situation in which: (a) benefits and burdens in society are distributed according to a set of allocation principles where the basic rights and needs of individuals and groups are considered and respected (distributive element); (b) the rules and regulations that govern decision making preserve basic rights, liberties, and entitlements of individuals, groups, or communities (procedural element); and human and other biological beings are treated with respect and dignity by all parties involved (interactional element) (Jost and Kay, 2010). Likewise, equality, which we use here in its opposite, conveys an ideal state of perfectly balanced or even distribution of goods and services across populations, while equitable can allow an element of self-determination. In a neo-liberal conception, as long as each member or group has an equal chance to obtain access to resources and options, a distribution can be termed equitable because it is self-determined on an equal playing field. Such equitable distributions are seen in this conception as fair or just because no one has had an advantage in gaining access to resources and options (Ikeme, 2003; Hughes, 2013). However, this conception ignores the power of assets and options, once attained by some individuals and groups, to create or compound differential access a to assets and options for others thus creating social inequality (Agyeman et al., 2016). Social inequality thus creates self-feeding systems that are not fair or equitable because they deny, to marginalized people and groups, access to assets and options necessary to avoid risks at the same they deny access to police systems and institutional features that could help them gain access those assets and options.

Inequality determines differential location and access to places, water, food, energy resources, and decision-making options in a city where resources are distributed unevenly across populations (Reckien and Lwasa, 2017). Typically such uneven distributions result from markets, power, other institutional mechanisms and risk mitigation and adaptation policies that engender or perpetuate socially defined categories of wealthy or poor, or of included and excluded populations (Stein, 2011; Romero-Lankao et al., 2016b) based on class, caste, gender, profession, race, ethnicity, age, and ability (real or perceived).

Undergirding our analysis in this paper is an assumption that, in the context of city climate action, an understanding of how inequality creates differences in exposure and vulnerability is fundamental to creating fair and effective risk mitigation and adaptation. Policies aimed at creating risk-equality should contain mechanisms to ensure the fair distribution of risks of negative impacts and of benefits (assets and options) to undertake climate action across city populations (distributive justice). Creating equality also means generating equal opportunities for participation and recognition for all, including underrepresented groups (procedural justice) (Bulkeley et al., 2013; Hughes, 2013; Reckien and Lwasa, 2017).

Among the resources and options that vary with inequality to create differential urban vulnerability, access to food, energy, and water are so basic and primary that they can be used as bellwethers of an uneven distribution of many other resources conditioning vulnerability Biggs et al., 2015; Romero-Lankao et al., 2016b. When considering the fair distribution of resources, assets and services related to distributive justice, it is important to recognize that differences in gender, race, socioeconomic status, and culture are part of procedural barriers that condition participation in policies affecting distribution. Thus, a cultural value can inhibit poor and marginalized populations from effectively participating in decisions (e.g., where to locate infrastructural investments in water and electricity) that affect their wellbeing, property, resources, climate risks, and capacities to adapt and mitigate.

## Conceptual Framework

Using discourse analysis, we qualitatively unpack how, in their adaptation plans, city officials' frame inequality in urban climate risk. We then combine discourse analysis and adaptation analysis to examine some of the issues addressed by the adaptation actions suggested in the plans. Lastly, we use a meta-analysis approach to quantitatively investigate patterns of framing and adaptation action across cities.

We will map narrative understandings in the adaptation plans of how inequality creates differences in exposure and vulnerability. We will also examine if and how adaptation actions contain mechanisms to ensure the fair distribution of assets and options to manage climate risks (distributive justice), and generate equal opportunities for participation and recognition for all, including underrepresented groups (procedural justice).

### Discourse Analysis

Various strands of social science scholarship have used discourse analysis to examine texts, images, papers, books, and reports to define the ideas and concepts—which we will call understandings—through which actors understand and act upon the world (Foucault, 1972; Sharp and Richardson, 2001; Hajer, 2004; Keller, 2011; Wiegleb and Bruns, 2018). Rather than being neutral, these narrative understandings privilege some socio-environmental facts and may suggest some policy actions over others (Sharp and Richardson, 2001; Hajer, 2004; O'Brien et al., 2007; Trombetta, 2008). We draw on section Conceptual Framework and on the Sociology of Knowledge Approach to Discourse to map the discourse of 43 adaptation plans (Keller, 2011). The sociology of knowledge analysis of discourse includes three components: knowledge structuring, discourse production, and power effects. Here we will only focus on the first and the third. We excluded the second, which entails an examination of the influence of sociopolitical context on framing and action (Keller, 2011), because our study focuses on discourse as it crystallized in the plans, and not on the influence of each city's sociopolitical context on framing and action.

To help us determine *knowledge structuring*, we mapped, through their references to issues of concern, the general interpretative frame city officials use to make sense of a climate change issue in their adaptation plans. For instance, do city officials frame climate adaptation as a problem of risk, or of resilience? However, setting issues such as those related to inequality in climatic risk on the adaptation agenda also relates to the way in which city officials determine what kind of problem climate change is. What causal SET*EG* factors are involved in the creation of climate change impacts? Are these impacts only the result of location and geography, or exposure? Or are they also the result of prior policies and unequal patterns of development determining differences in the vulnerability of people and FEW-systems within cities?

Drawing on the discussion of existing literature (section Conceptual Framework), we will map how adaptation plans address inequality in hazard exposure and in the following multiscale (SET*E*G) factors determining vulnerability (Arup, 2014; Romero-Lankao and Gnatz, 2016).

- Locational (exposure) factors conditioned by the presence of populations and critical FEW infrastructures in places that could be adversely affected by floods, heatwaves, and other climate hazards (Nicholls et al., 2008).
- *Socio-demographic* factors consist of age, gender, and demographic structure of a city or the behavior of individuals and groups (Donner and Rodríguez, 2008).
- *Economic* factors relate to uneven economic growth, urbanization, income, and affordability of food, energy, water, and other resources (Uejio et al., 2011).
- *Techno-infrastructural* and built environmental factors include land use change and the distribution, quality, and robustness of water, sanitation, electricity and related, FEW critical infrastructures, and systems. Critical FEW infrastructures include electric power, natural gas and oil, water supply, and food distribution systems, but because we acknowledge the role of transportation, telecommunications, health, emergency and other services, we also included these as critical urban FEW infrastructural systems (Rinaldi et al., 2001).
- *Environmental* factors such as the biophysical and climatic characteristics affecting an urban area's predisposition to hazards relate to exposure. For instance, coastal cities are prone to sea level rise, storm surge and coastal flooding, saltwater intrusion and tropical storms.
- *Governance* factors consist of the fit between areas of concern and authority, cooperation, and cohesiveness among governing bodies and levels of government, policies and actions, and the legacies of actions and policies around-land use planning; and through investments, location and climate proofing of FEW infrastructure and service networks, which shape the geography of urban risk (Aylett, 2014).

*Power effects* relate to the intended or unintended consequences emerging from the discourse. Elements of the power effects include the *dispositifs*, a French word describing the institutional, organizational and infrastructural elements, which we define here following Foucault and Keller as the suggested apparatuses of adaptation action, such as

a) Personnel and organizations charged with undertaking adaptation policies;
b) Institutional and organizational processes seeking to evaluate, monitor and understand the climate change problem, or to foster awareness among city actors, decision makers, and populations. We will include these under institutional-behavioral adaptation actions (note that (a) and (b) seek to address the sociodemographic and governance factors within our SET*E*G framework);
c) Investments in and climate proofing of critical FEW infrastructure (artifacts), which we will include under techno-infrastructural actions. (These address the techno-infrastructural factors within our SET*E*G framework); and
d) Other discursive or non-discursive adaptation actions, such as environmental and economic adaptation actions (which address respective factors within our SET*E*G framework).

Such “*dispositifs*” are shown in the literature to hold the potential to address climate risk to people and FEW-systems in cities. In our analysis we sort “*dispositifs*” among techno-infrastructural, institutional-behavioral, economic, and environmental action categories (Romero-Lankao et al., 2017b).

### Adaptation Analysis

We also include insights from the climate adaptation literature to add accuracy to our discourse analysis. In the climate adaptation literature, institutional-behavioral actions include changes in the procedures, incentives, or practices of city actors, and often work through existing urban competencies and hybrid actor arrangements in sectors, such as urban planning, health, water, energy, and disaster risk management (Fisher, 2013; Romero-Lankao et al., 2017b). Institutional behavioral actions entail the creation of organizations charged with mainstreaming adaptation into other sectoral and developmental policies such as urban planning, transportation, and disaster management; with evaluating, monitoring and understanding the climate change problem; and with fostering awareness among city decision makers and populations. In the environmental justice literature, these actions are fundamental to procedural justice by broadening participation in, recognition, and commitment to adaptation across governmental, private, civil society, and community actors (Bulkeley et al., 2013; Shi et al., 2016; Reckien and Lwasa, 2017).

Techno-infrastructural actions are critical in the creation of artifacts, such as energy, water and sanitation. They are often framed in the climate adaptation literature, as efforts to discourage growth in risk-prone areas and to protect critical urban infrastructural systems through investments in climate proofing, and changes to design, operational, and maintenance practices (Romero-Lankao et al., 2017b).

Other adaptation actions include economic and environmental policies. The former aim at creating enabling conditions for autonomous action by governmental and nongovernmental actors, and to support broader development goals. Funding programs from public and private sectors are fundamental. By strategically allocating funding (whose amount and sources vary widely across cities), local governments can effectively respond to climatic risks (Aylett, 2014). Environmental *actions* seek to manage the biophysical, climatic, and hydrological factors affecting an area's predisposition to hazards (Brink et al., 2016; Kabisch et al., 2016). Environmental actions take into account and manage the role of biodiversity, greenspaces, and other ecosystem services in mitigating hazard risk and reducing the vulnerability of urban populations and FEW systems to climate change (Levy et al., 2014).

## Study Design

Meta-analysis is often applied to find commonalities within a variety of research papers and methods (Littell et al., 2008). It involves the pooling of data that quantitatively examine whether causal relations described in individual papers (e.g., drivers of climate risk, determinants of vulnerability of food, energy, and water insecurity) hold across a broader body of scholarship (Misselhorn, 2005; Romero-Lankao et al., 2012).

While meta-analysis is frequently combined with systematic literature reviews to synthesize the results of previous research, in our approach, we combine meta-analysis with discourse analysis to systematically investigate patterns on the framing of inequality in risks within a selection of 43 adaptation plans.

### Selection and Analysis of the Adaptation Plans

This study resulted from a prior report commissioned by the C40. Although the C40 has 94 affiliated cities, we only got access to 60 adaptation plans for analysis. Of these, we selected 43 plans, 4 of which are from cities located in lower-income, 12 in middle-income and 27 in upper-income countries. As can be seen in Figure 1, our selected sample also has a good representation of C-40 cities from Latin America, Europe, North America, Africa, and South-East Asia.

We built on our prior work on FEW nexus, climate adaptation and inequality cited in section Conceptual Framework, and on the review of the adaptation plans, to map how city officials prioritize policy actions to manage inequality in risk. Although we couldn't analyze how individual city officials actually understand the climate change adaptation and FEW issues we studied, we did analyze the understandings of these issues conveyed in the plan. We will refer to these understandings, conveyed in the plans, as narrative understandings.

Our data extraction and synthesis followed an examination of discourses and a meta-analysis approach (Littell et al., 2008; Keller, 2011; Romero-Lankao et al., 2012; Wiegleb and Bruns, 2018). Our conceptual framework functioned as a starting point to design and test a review template and to agree on our own definition of terms and fields (available upon readers' request). We then used this template to extract data from each of the 43 adaptation plans. First, each selected plan was carefully reviewed by at least two members of our research team to ensure systematic and consistent data extraction. Factors influencing risk to people and FEW-systems were identified and coded into the five SET*E*G domains (i.e., sociodemographic, economic, techno-infrastructural, environmental, and governance). Adaptation actions were classified into institutional-behavioral, techno-infrastructural, economic, and environmental.

We further subdivided these categories of SET*E*G factors and adaptation actions into terms, as described in the second column of Supplemental Tables 1A,B, 2A–E, 3A–D). After summarizing each adaptation plan, mention counters were developed, based on mention of the terms, to capture overall narrative understanding (Supplemental Tables 1A,B, 2A–E, 3A–D). Once a term was found, the counter maxed at “1” for that particular topic to avoid duplicate counting. Limiting mention counts to one per plan is the most effective way to avoid bias in answering the question: what plans address what topics? Although this method does not seek to answer what plans emphasized what topics. It does answer the question what issues were emphasized in the plans overall. We use two approaches to refer to the percentages:

1) Number of plans with mentions of an issue/total number of plans
2) Number of mentions of an issue/total number of mentions of all issues within a category

The first gives a view of the relative importance, attributed by urban policymakers, to particular issues within plans compared with all plans. The second gives a view of the relative importance, attributed by urban policy makers, to particular issues compared to all issues within a given category (e.g., techno-infrastructural vs. institutional-behavioral actions). Together, these measures give a two-scoped view of the relative priorities given by urban policymakers to the issues addressed in the plans.

Although we feel this study offers many relevant insights, it was faced with some constraints that may affect its outcomes. While we included 43 cities from low-, middle-, and high-income countries, these were not selected using a sampling approach. Due to our determination to have at least two members review each plan, and our group's language limitations, we could only review plans written in English and Spanish. This meant we were not able to analyze the discourse in many plans that might have offered additional insights. Readers of this paper should, therefore, keep in mind that while the combination of discourse analysis with meta-analysis to identify patterns in understanding and action is innovative, our study is exploratory in nature. Furthermore, while our use of a discourse analysis to examine the framings of inequality in risks exposed some of the narrative understandings conditioning policy actions, it did not include an examination of why and how the socio-political and geographical contexts in which city officials operate shape their interpretations and planned actions. Lastly, since we studied plans and not implementations we could not determine how (or if) the suggested adaptation actions were implemented.

While ethical questions regarding this study might be raised around the fact that it was commissioned by the C40 to study the adaptation plans of C40 cities, giving rise to concerns about scientific objectivity, we feel that our analysis of these plans was objective and sound for two reasons: (1) We studied the adaptation plans as independent documents and not as they pertain to the C40 or its mission; and (2) The methods used in the study were evenly applied across city adaptation plans without regard to any city's membership, income level or status in the C40.

## Narrative Understandings and Policies in the Adaptation Plans

This section is organized around three topics. The first and second include a mapping of the narrative understandings—or knowledge structuring—crystallized in the adaptation plans. This not only in terms of what interpretative frame is used but also in terms of what locational and SET*E*G factors are identified as key determinants of climate risk, and whether inequality is considered in this conveyed understanding. The third topic refers to the power effects in the form of adaptation actions suggested in the adaptation plans to address inequality in risk to people and FEW-systems.

### Interpretative Frames

We found that the urban adaptation plans analyzed here embed adaptation in a larger vision for the city, often with a multiplicity of coexisting frames. Many of these interpretive frames are not only full of symbolism, as in the resilience framing we will describe later in this section, they also feature key—and sometimes, contradictory—organizing principles of policy action (Figure 2). Rather than converge toward an integrated understanding, these concepts often coexist in a tension of incongruent and unbalanced sets of principles and related actions. In this disharmony, economic and investment concerns and interests (e.g., infrastructural and economic investments) tend to take precedence over concerns and interests for the environment and the marginalized (see next subsection).

Frequently cities appear in the adaptation plan narratives as leaders, development hubs or engines of innovation and investment, key to growth and stability nationally, and internationally. Adaptation in this context forms part of a broader sustainability vision present in many cases for the creation of a vibrant, economically prosperous, and socially just cities, or cities that are habitable, secure, resource-efficient, socially and economically inclusive, and competitive internationally (Seattle, Tshwani).

In many adaptation plans, city officials frequently see climate change as posing risks, but also offering opportunities. These include opportunities to attract investment, generate high-value jobs, strengthen research and development, or foster circular or green economies. For instance, the Singapore plan states that the city is poised to tap economic opportunities offered by global warming, such as investments in new growth areas, the creation of high-value jobs, the promotion of green growth, and of R&D capabilities.

Interestingly, 87% or 37 of the reports apply a risk approach to frame climate change issues (Figure 2). Risk is often framed in the adaptation plans as the probability of occurrence of a hazard, such as sea level rise, multiplied by a consequence such as property damage. While differences in emphasis exist, a dominant narrative emerges, underlying the risk approaches in these plans. Common to this narrative is the idea that strategies for the protection of urban areas from the risks and FEW constraints associated with climate change require a scientifically grounded technical assessment of how changes in temperature, precipitation, and sea level are likely to affect critical infrastructures, resources and economic activities in the cities.

Adaptation plans reviewed in this study illustrate that resilience is, increasingly, becoming embedded in the discourses of urban decision-makers. Resilience is not only seen in the plans as an ecological principle, but also, frequently, as an opportunity. Such opportunities, when coupled with appropriate actions, can increase a city's economic, energy, environmental, and food security, in addition to protecting the quality of life and safeguarding property (e.g., Durban). It is, therefore, common for the adaptation plans to frame the hazards and disruptions brought about by climate change as somewhat of a blessing in disguise. In this discursive thread, cities may even view themselves as symbolically endowed with a power of resilience like “the mythic phoenix,” able to take advantage of disruptive events and carry on through challenges over the years. In such cases cities become a phoenix aware of how the threats cities face—and their responses to these threats— expose several interdependencies that city officials must better comprehend (San Francisco). An almost mythic idea of its own resilience can also be found, for instance, in the New Orleans plan, which describes a city certain that the creativity and resilience of its people and places have been key in its capacity to bounce forward, after being faced with a decade of hurricanes, oil spills, and the Great Recession.

### Inequality in Climate Risk

**Figure 3:**
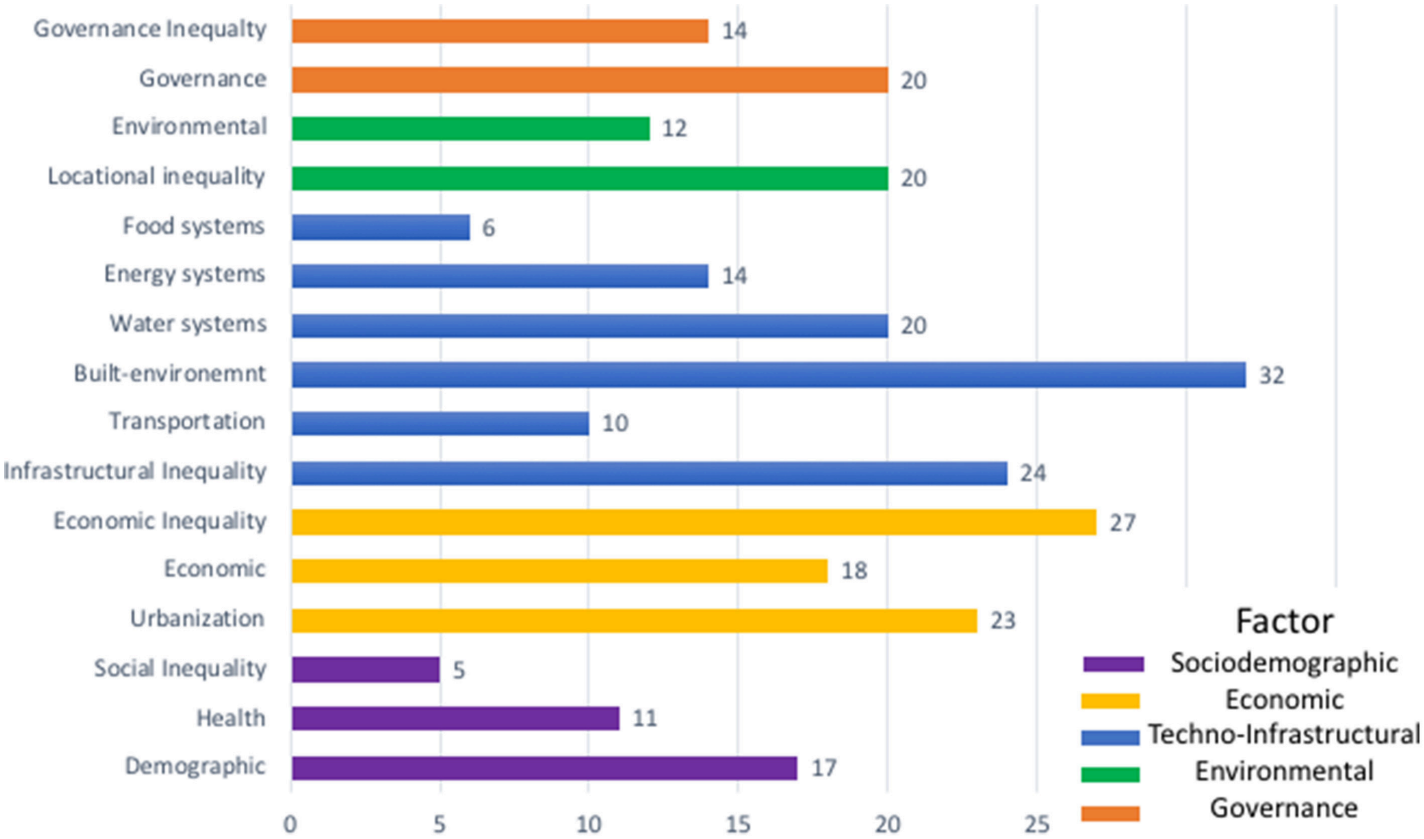
Risk factors receiving attention in the adaptation plans. After reading and summarizing each adaptation plan, we subdivided the risk factors into five SETEG domains (marked by different colors above) and related factors (within each color). For more details, see Supplemental Tables 2A–E.

We compared levels of attention paid to climate risk associated with five selected SET*E*G factors, and examined whether the plans mentioned inequality in reference to these factors (inequality within each domain, Figure 3). This comparison revealed that because city officials are, by necessity, generalists, adaptation plans deal with many climate change issues at a time, from those related to economic development and land tenure to those associated with health, disaster management, housing and critical FEW infrastructures (Supplemental Tables 2A–D).

Evidence from the narrative understandings conveyed by the plans suggests that FEW-nexus thinking is not yet embedded in city officials' priorities, or that such considerations create a conundrum that officials are reluctant to tackle. Of the total of risk factors, those related to food, energy and water systems were mentioned in 6, 14, and 20 reports, respectively (Figure 3). Where they did appear, food, energy, or water systems are treated separately, in most cases, without consideration of how their interdependencies can amplify or mitigate risk. The influence and vulnerability of FEW-systems was often framed in terms of techno-infrastructural issues associated with age, design or capacity characteristics (Blue bars, Figure 3). For example, the plans mention that FEW-systems and infrastructures are vulnerable because they are old, designed without consideration of the new (and unstable) normal that climate change will bring, and in need of retrofitting and climate-proofing actions. Buildings are also vulnerable because of poor quality design and construction, age, and lack of maintenance (Figure 3; Supplemental Table 2C). Inequality also tends to be given a lower priority and appears mainly in relation to other factors and very rarely in relation to FEW systems.

Inequality considerations were included in 24 plans and represented 26 percent of the total mentions of *techno-infrastructural* risk factors. However, scant consideration was given to how techno-infrastructural and built environment factors condition unequal risk through such distributive mechanisms as differential access to water or sanitation, or differences in the provision and placement of infrastructures and services such as electricity, waste disposal, tree shading, parks, hurricane shelters, and evacuation routes.

Locational (*exposure*) factors were mentioned in 32 plans (green bars, Figure 3) as related to differential exposure of populations and FEW-systems to climate hazards. Adaptation plans in Peru, Mexico City, and Cape Town point to how the poor are priced out of desirable neighborhoods and are often forced to live in hazardous areas. In Seattle, San Francisco, and New Orleans, adaptation plans show concerns for how inequality makes poorer populations more likely to occupy low-lying areas, prone to flooding or more likely to experience heat island effects because these areas are more affordable.

Related to location, *environmental* risk factors were mentioned in 12 plans (green bars, Figure 3). Some of these mention that many informal settlements locate on areas, where the high-water table and inadequate infrastructure make them particularly vulnerable to flooding (e.g., Cape Town, Buenos Aires, Tshwane, Mexico City, and Lima). Cities from the Global North also offer examples of how low-income communities living in brownfields or in flood risk areas face higher levels of exposures not only to sea level rise, floods and heatwaves but also to contaminated land (e.g., New York, and New Orleans).

Regarding *economic* factors, twenty-seven adaptation plans (67%) refer to economic development as a key determinant of risk, and twenty-three (53%) of all plans mention urbanization as a broader driver of risk (yellow bars, Figure 3). Interestingly, 27 or 62% of the adaptation plans referred to unequal economic growth conditioning access to determinants of a population's capacity to mitigate risks and to adapt. Such determinants include location, and access to secure land, affordable, accessible, and good quality housing, energy, water, food, and transportation (yellow bar, Figure 3).

In the adaptation plans of Lima, Mexico City and Cape Town, the narratives acknowledge deep inequalities and high poverty rates that relate to the existence of informal, unplanned settlements whose populations have precarious housing without adequate FEW resources necessary to protect themselves against hazards. Recognition of such conditions is rare in the adaptation plans of the global north. New York is one of the handful of such cities indicating that nearly half of its people live in or near poverty, and lack access to good quality housing and other resources needed to adapt.

While 17 adaptation plans refer to *socio-demographic* factors such as population size and growth, age, gender, and pre-existing medical conditions as determinants of vulnerability, 20 plans convey an understanding of governance as a determinant of risk and vulnerability (purple bars, Figure 3). Such governance conditioned risks operate through investments and the location of FEW infrastructures and service networks, and through the legacies of actions and policies around-land use planning or its lack though this is not generally acknowledged in the plans (orange bars, Figure 3).

As for inequality*, socio-demographic* and *governance* factors, creating social exclusion by class, gender, race, migration, and minority status were mentioned in 13 and 5 plans, respectively, (orange and purple bars, Figure 3). Adaptation plans from cities in middle- and low-income countries tended to mention the influence of social exclusion on inequality in access to affordable energy, water, food, and sanitation, and reliable transportation systems more often than plans from high-income countries. Race, however, appears in the adaptation plans of the US cities of New York, New Orleans and San Francisco as a predictor of risk. These plans indicate that people of color are more likely to live in areas more at risk of flooding and subsidence, to live in poverty, to be unemployed and to have pre-existing health conditions associated with higher hazard risks. These plans also recognize that their marginalized populations have lower capacities to mitigate and adapt (Supplemental Tables 3A–D).

### Policy Actions to Address Inequality in Risk and FEW-nexus

In our mapping of the *power effects* emerging from adaptation discourse among policymakers, we examined whether planned adaptation actions aimed at either reducing hazard exposure or tackling the drivers of social vulnerability considered inequality. The adaptation actions identified were organized into “*dispositifs*” as defined in section Tracing Existing Scholarship. We sorted “*dispositifs*” among techno-infrastructural, institutional-behavioral, economic, and environmental action categories.

Our findings suggest that, while proposed adaptation actions tend to target many issues at a time, they also tend to prioritize infrastructural and economic issues, and that inequality is a secondary concern. Furthermore, city officials tend not to address the links and feedbacks between critical FEW infrastructural systems but rather to suggest actions to manage each infrastructural system at a time.

**Figure 4:**
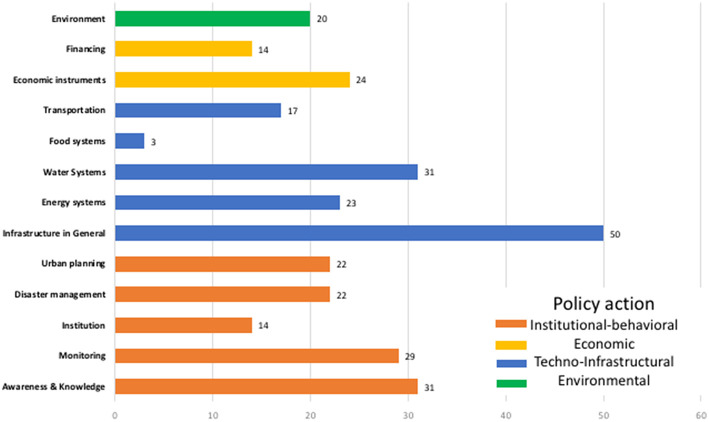
Policy action suggested in the adaptation plans. After reading and summarizing each adaptation plan, we subdivided the adaptation actions into four domains (marked on different colors above) and related factors (within each color). For more details, see Supplemental Tables 3A–D.

Technological-infrastructural actions, which can be a means of fostering distributive justice, received the highest number of mentions (with 124, or 41%, blue bars, Figure 4). However, by and large distributive justice was not considered. Instead, actions were presented in the plans as a means to protect buildings and infrastructure through changes to design. Similar to what we found in our examination of narrative understanding, suggested policy action did not address the links and interdependencies among critical FEW-systems but rather focused on one sector at a time. Examples of planned infrastructural adaptation actions included:

- Improving energy redundancy and reliability of (e.g., distributed power), flood fitting the design of surfaces, and increasing the extent of cooler, green surroundings (Changwon, Chicago, Karachi, New Orleans, Paris, Seattle).
- Introducing low-carbon or renewable energy sources, reducing coal usage for electricity generation, promoting energy-efficient and resilient technologies, appliances, and designs in buildings and developments—e.g., cooling systems, LED and fluorescent lighting (Amsterdam, Quito).
- Adapting water infrastructures to withstand heavy rain events, drought, and heat. Climate-proofing water systems and implementing a water sensitive approach to urban design and flood mitigation through blue and green infrastructures (Copenhagen, New York, Rotterdam, San Francisco).

Techno-infrastructural actions were most frequently organized around resilience, low-carbon utilities and buildings, promoting a circular economy, and risk as a source of investment opportunity (Supplemental Table 3A). For instance, Amsterdam and Boston suggested fostering a circular economy to reduce waste and increase recycling throughout economic activities and districts. Other cities, such as Copenhagen, suggested basing adaptation on a risk and resilience approach aimed at improving infrastructure adaptability to new or unexpected conditions by achieving a city-wide, multiple-purposed, and longer-term risk mitigation vision.

There were a few exceptions were plans used techno-infrastructural actions aimed at addressing inequalities in risk. For instance, the following actions were suggested:

- Reducing intra-urban differences in water scarcity, access and use; increasing water coverage to poor and informal populations without regular, safe, and continuous water service (Cape Town, Durban, Johannesburg, Kolkata, and Mexico City); and providing access to weatherization of homes to low income families (Seattle).
- Scaling up development tied to renewable energy services to accomplish a lower energy impact while achieving reduced poverty and promoting economic development (Durban, Tshwane).
- Fostering structural investments that consider the consequences from interrupted energy supply during and after extreme events, and target those that are more affected (Durban, Tshwane).
- Renovating slums, informal, or poor settlements (Addis Ababa, Buenos Aires, Cape Town, Durban, Kolkata, Mexico City, and Tshwane).

Institutional-behavioral actions were second in the number of mentions (118 or 39% of the total). The focus in order of importance was on knowledge and awareness, monitoring, urban planning, disaster risk management, and institution building (orange bars, Figure 4).

Awareness and knowledge, and monitoring were addressed in 31 and 29 of the plans, respectively. These plans suggest a suite of strategies to systematically evaluate, assess, understand, and monitor the kinds of climate risks and vulnerabilities they face (Supplemental Table 3B). They also suggest using scientific and technical expertise as a vital source of knowledge. For instance, Amsterdam suggests improving the city's knowledge and understanding of data to become active partners, steering events toward sustainability based on a knowledge of interconnections between systems such as energy and water.

Two crucial adaptation instruments received attention in 22 adaptation plans each: disaster risk reduction (DRR) and urban planning. Elements of DRR included early warning systems, cooling centers for poorer populations, and climate-sensitive management protocols (e.g., Bogota, Kolkata, Mexico City, San Francisco, Quito, Rio De Janeiro, and Sydney). Urban planning was mentioned as a fundamental tool for anticipating climate change impacts, fostering early action and even preventing risks (orange bars, Figure 4). Some plans (e.g., Lima and Tshwane) acknowledged institutional barriers to effective implementation, such as weak law enforcement. Others pointed to gaps in the levels of authority and autonomy to control the investments and decisions that are fundamental not only for effective urban planning but also for managing the drivers of climate risk in the city.

FEW thinking with relation to equality received scant attention within planned institutional-behavioral actions. We found only the following few examples of strategies to enhance equality within each sector:

- Community based adaptation actions such as upgrading informal settlements, building flood-water drainage, and sewer systems in poor areas (Mexico City and Tshwane), and training poor communities for the management and attention of disasters (Bogota).
- Increasing the share of renewable energy per capita through demand management actions, such as agreements with a number of utilities, incentives that support energy efficient practices, and reduced electricity consumption during peak hours (Amsterdam, Durban).
- Inducing water conservation through water restriction, tariffs, and reduction of leaks (Cape Town).
- Enforcing polices and by-laws that make healthy food accessible to all (Boston) and reserve space for local decentralized food hubs that can supply small traders while reducing ecological impact, through the support of small scale, sustainable farming practices (Durban).

Within the economic instruments suggested in 38 adaptation plans, equality considerations were, likewise, virtually absent. While many of the plans seek to create enabling environments for independent action by both governmental and non-governmental actors, for example through infrastructural investments, they largely aim at enhancing their economies without regard for structural inequality or uneven distribution. Through these actions, the plans also aim to support broader goals such as the Sustainable Development Goals. Indeed, the governments that produced many of the adaptation plans we analyzed are driving investments in major flood defenses, and in the transportation, water, and sanitary services sectors, but generally steer away from equality considerations in these investments and are more concerned with how they will fund them. Some cities, particularly from high-income countries, are explicitly and actively partnering with the private sector (Amsterdam, Copenhagen). One of these plans acknowledges that society at large will pay a large dividend to have infrastructures privately constructed and operated (Copenhagen).

Environmental actions were considered in 40% of the plans, and many of these contain actions primarily focused on increasing or protecting biodiversity (e.g., Karachi, Montreal, Seoul, and Los Angeles), and on strategies for managing ecosystem services (green bar, Figure 4 and Supplemental Table 3D). For instance, the plans suggest actions to green the cities' streets, parks, and open spaces in order to serve multiple risk mitigation purposes. Other planned actions include efforts to increase biodiversity and reduce the urban heat island effects (e.g., Sydney, Vancouver, Melbourne), to increase urban agriculture (Seoul), and to better manage such hazards as runoff or fires (e.g., Rotterdam, Melbourne, Rio de Janeiro, and Portland). Nature- or ecosystem-based adaptation actions are also suggested to increase the resilience of vegetation to climatic and ecological impacts (such as erosion, Montreal), or to establish temporary rainwater catchment systems (Mexico City). Some cities also suggest conservation or rehabilitation of degraded ecosystems (Tshwane, Quito, and Mexico City) and protecting or restoring natural protections in coastal areas (New Orleans).

## Adaptation Plans and Risk Inequality

In this study, we examined evidence from 43 adaptation plans to determine whether and how they considered the factors driving inequality in exposure and vulnerability of people and the FEW systems that support them. To do this, we combined a discourse analysis with a meta-analysis of adaptation plans for 43 C40 cities. We are not the first scholars to conduct metanalysis. Examples of existing literature include (Misselhorn, 2005; Romero-Lankao et al., 2012; Endo et al., 2015). Nor are we the first to examine environmental discourse, even with regard to FEW systems. For instance, existing discourse scholarship has shown that a risk approach is prevalent among FEW nexus scholars (Wiegleb and Bruns, 2018). Because risks lack immediacy—says the analysis—discourse around FEW risks entails connecting a future scenario to a policy, “presented as a way of preventing that risk from materializing into real harm” (Corry, 2012. p. 244).

Our methodological innovation lies, rather, in our combination of discourse analysis with meta-analysis. We used this combination to examine narrative understanding and planned adaptation actions in 43 city adaptation plans. We integrated several theoretical strands of scholarship, such as FEW-nexus thinking, adaptation, and inequality, climate change risk, and adaptation and discourse analysis. Nevertheless, we did not examine why and how the socio-political and geographical contexts, in which city officials operate shape their interpretations and planned actions. Nor were we able to determine how or if the suggested adaptation actions were implemented. These represent the short-comings and limitations of our study that make it largely exploratory in nature. Notwithstanding these limitations, however, some clear patterns emerged that can help guide future research and policy.

We found that FEW-nexus thinking is not yet embedded in city officials' narrative understandings of risk and planned adaptation actions, even when unpacking interdependencies among food, energy, and water systems may help cities tackle some of the root causes of vulnerability and risk (Romero-Lankao and Norton, 2018). Other scholars have already pointed to the fact that, while promising, FEW-nexus thinking faces many practical challenges. For instance, knowledge integration is constrained by the existence of a plurality of sectors, levels of government, power, values and ways of understanding and managing climate risk (Leck et al., 2015; Romero-Lankao et al., 2017c). Scholars also suggest that local governments lack the institutional and organizational capacities needed to appropriately manage the complexity and uncertainty associated with climate risks, let alone inequalities in the vulnerability of people, or how that vulnerability interplays with FEW systems. Officials within sectors involved in managing climate risk, such as food, energy, water, disaster risk management, and urban planning hold diverse organizational and cultural values. They lack the incentives, rights, financial resources, and responsibilities needed to work across sectors and jurisdictions (Scott et al., 2015). Additionally, decision makers involved in DRR and adaptation policies lack interaction and coordination because of differences in language and political culture (Schipper, 2009). An examination of these factors is an essential first step to develop the skill sets, tools, funding, and incentives needed to foster nexus thinking in risk mitigation and adaptation practice.

In the city adaptation plans we analyzed, we found multiple frames coexisting behind the broader adaptation visions conveyed in their narratives. Rather than converging, issues and principles such as those of equality, coexist with economic issues in an imbalance of incongruent political movements and priorities (Anguelovski and Carmin, 2011; Campbell, 2013). In this disharmony, techno-infrastructural and economic investments and concerns tend to take precedence over concerns and interests for inequality or the environment in climate risks.

Clearly, challenges exist with under-investments, backlogs and deferred maintenance of infrastructure. Urban infrastructures in many developed countries are deteriorating, and in developing countries infrastructure construction and maintenance have often failed to keep pace with the dynamics of urbanization (Kraas et al., 2016). Adaptation plans recognize that by working as a risk amplifier, climate change is projected to intensify these challenges, through at least two mechanisms: long-term, slow impacts such as constant deterioration of storm water system due to floods mentioned in the adaptation plans of 27 cities, or extreme events such as hurricanes (mentioned by 10 cities) and damaging critical FEW infrastructural systems.

Still, with a few exceptions, equality concerns were not the priority. In the adaptation plans, narrative understanding and policies to address techno-infrastructural challenges were frequently organized around resilience, low-carbon utilities and buildings, promoting a circular economy, and risk as a source of investment opportunity. All these strategic decisions advance cities as centers of economic and infrastructural growth. However, they run the danger of fostering inequality in access, related to distributional justice, by creating climate proof places that become more exclusive and expensive, pricing out marginalized populations who end up living in less desirable areas and lacking access to critical FEW infrastructures (Coutard, 2008; Zérah, 2008).

In their adaptation plans, cities of high-income countries are seeking to explicitly and actively partner with the private sector (Amsterdam, Copenhagen). Policy-makers in these cities reason that moving infrastructural development and operation to the private sector can be a way of diverting development costs away from government and reducing the need for politically unpopular taxes. However, this hasn't often shown itself to be a good strategy, as private interests must inevitably draw profits from their projects, leaving less for the public good. Ultimately, this will have implications for inequality in risk, as the poor communities, those most in need of investments in climate proofing, are more likely to be excluded not only from decisions (procedural justice) but also from reaping the benefits of techno-infrastructural interventions (distributional justice) (Coutard, 2008; Zérah, 2008; Revi et al., 2014).

Socio-institutional actions relate to the distributive and procedural aspects of equality in different ways (Reckien and Lwasa, 2017). For instance, by involving vulnerable populations in decisions on land use and location of infrastructural investments, in the generation of knowledge, or in the monitoring of climate risks (Moser, 1998; Moser and Satterthwaite, 2010; Bouzarovski, 2014). Nonetheless, rather than using participatory instruments such as community based adaptation (Ebi and Semenza, 2008; Dodman and Mitlin, 2013), the plans mostly suggest using scientific and technical expertise as a vital source of knowledge. There are reasons for this. Climate change adaptation is highly data-dependent, demanding that city officials engage in new ways of gathering data, collaborating with scientists, using scientific information, and dealing with uncertainty (Hughes and Romero-Lankao, 2014). Yet, the focus on technical knowledge is a key element of prevalent cultural values that inhibit poor and marginalized populations from effectively participating in decisions on where to locate FEW critical infrastructural investments that affect their well-being, property, resources, climate risks, and capacities to adapt and mitigate. Although our current study, based purely on textual analysis, did not attempt to examine socio-political context (knowledge production), our conclusions do suggest that sociopolitical context was at play in the creation of the plans. Even beyond that, they suggest that common elements in socio-political context may be drawing cities away from actions based on effectively addressing such complex concerns as vulnerability and inequality toward those least conflicting with economic priorities.

The relatively low importance of equality considerations in the adaptation plans will likely limit the capacity of cities to support broader goals such as the Sustainable Development Goals, Sendai Protocol for Disaster Risk Reduction and New Urban Agenda (Simon et al., 2016). The purposefully inclusive scope of the New Urban Agenda and of the targets and indicators in the urban SDG (Goal 11) provide a unique opportunity to include equality considerations in adaptation (Romero-Lankao et al., 2018). Prospects for progressing and mainstreaming climate change agendas, therefore, depend on demonstrating that climate agendas do not always and irreconcilably conflict with development priorities, such as those related to equality. From a longer-term perspective, they are essential and complementary to them.

## Author Contributions

PR-L led the design, gathering, analysis and interpretation of data for the work. She also drafted and revised the work critically for important intellectual content. DG contributed to the design, analysis and interpretation of data for the work. He also drafted and revised the work critically for important intellectual content.

### Conflict of Interest Statement

The authors declare that the research was conducted in the absence of any commercial or financial relationships that could be construed as a potential conflict of interest.
