# Supplementary material for: Risk Inequality and the Food-Energy-Water (FEW) Nexus: A Study of 43 City Adaptation Plans
Source: Front Sociol. 2019 Apr 18;4:31. doi: 10.3389/fsoc.2019.00031 (PMC8022741; doi:10.3389/fsoc.2019.00031)
Supplement: Supplementary file 1 [file Data_Sheet_1.docx]

**Table 1A: Adaptation Plans by City, Country, Income-Level, Year of Publication and Title**

Table 1B

| **Broader Vision** | | | |
| --- | --- | --- | --- |
|  | Term | Mentions | Total Mentions |
| Risk | disaster risk reduction | 5 | 37 |
|  | mitigation | 20 |  |
|  | adaptation | 28 |  |
|  | hazard | 7 |  |
|  | impact | 8 |  |
| Economic  Growth | green economy | 3 | 14 |
|  | economy | 7 |  |
|  | sustainable development | 6 |  |
|  | carbon neutral | 1 |  |
|  | green growth | 4 |  |
| Leadership | leadership | 4 | 12 |
|  | hub | 3 |  |
|  | collaboration | 1 |  |
|  | leader | 9 |  |
| Resiliency | resilience | 10 | 11 |
|  | resiliency | 2 |  |

Note: After reading and summarizing each adaptation plan, we identified cities’ framing of adaptation. Within each of these, we developed mention counter to capture the approach emphases of the narratives. While “mentions” count the number of times each plan mentions a term, in "total”, if two or more terms are mentioned in the same plan, they only count as one. This removes the problem of double counting.

**Table 2A: Exposure Factors Receiving Attention in the Adaptation Plans**

| **Locational (exposure)** | | | |
| --- | --- | --- | --- |
| Factor | Term | Mentions | Total |
| Physical- Environmental | flood line | 1 | 12 |
|  | floodplain | 2 |  |
|  | air quality | 1 |  |
|  | landslide | 1 |  |
|  | trees | 1 |  |
|  | park | 1 |  |
|  | subsidence | 1 |  |
|  | SLR | 1 |  |
|  | low-lying | 2 |  |
|  | coastal | 2 |  |
|  | flood | 12 |  |
|  | unstable hill | 1 |  |
|  | active ravine | 1 |  |
|  | marginal land | 1 |  |
|  | drainage channel | 1 |  |
|  | wetland | 1 |  |
|  | river | 2 |  |
|  | mountain | 1 |  |
| Socio- economic | population | 12 | 20 |
|  | of color | 4 |  |
|  | poor | 7 |  |
|  | informal settlement | 4 |  |
|  | informal communities | 1 |  |

Note: After reading and summarizing each adaptation plan, we identified the exposure factors, and subdivided them into related terms. Within each of these, we developed mention counters to capture the emphases of the narrative. While “mentions” count the number of times each plan mentions a term, in "total", if two or more terms are mentioned in the same plan, they only count as one. This removes the problem of double counting.

**Table 2B: Sociodemographic Factors Receiving Attention in the Adaptation Plans**

| **Sociodemographic** | | | |
| --- | --- | --- | --- |
| Factor | Term | Mentions | Total |
| Demographic | population | 12 | 17 |
|  | demographic | 1 |  |
|  | aging population | 1 |  |
|  | young | 3 |  |
|  | aged | 2 |  |
|  | children | 5 |  |
|  | elderly | 7 |  |
|  | women | 1 |  |
|  | education | 1 |  |
| Exclusion | underprivileged | 1 | 11 |
|  | of color | 4 |  |
|  | migrant | 2 |  |
|  | refugee | 1 |  |
|  | racial disparities | 1 |  |
|  | disadvantaged | 1 |  |
|  | marginalization | 1 |  |
|  | minority | 1 |  |
|  | minorities | 1 |  |
|  | living alone | 1 |  |
|  | isolated | 2 |  |
| Health | disabilities | 1 | 5 |
|  | disable | 2 |  |
|  | health inequalities | 1 |  |
|  | pre-existing health | 1 |  |
|  | healthcare | 1 |  |

Note: After reading and summarizing each adaptation plan, we identified the sociodemographic factors, and subdivided them into related terms. Within each of these, we developed mention counters to capture the emphases of the narrative. While “mentions” count the number of times each plan mentions a term, in "total", if two or more terms are mentioned in the same plan, they only count as one. This removes the problem of double counting.

**Table 2B: Economic Factors Receiving Attention in the Adaptation Plans**

| **Economic** | | | |
| --- | --- | --- | --- |
| Factor | Term | Mentions | Total |
| Urbanization | population | 11 | 23 |
|  | urbanization | 9 |  |
|  | growth | 9 |  |
|  | urban sprawl | 1 |  |
|  | development | 4 |  |
|  | Informal | 4 |  |
|  | settlement | 2 |  |
|  | land use | 2 |  |
| Economic | job | 4 | 18 |
|  | economy | 3 |  |
|  | economic | 7 |  |
|  | income | 4 |  |
|  | affordability | 1 |  |
|  | import | 1 |  |
|  | power | 3 |  |
|  | expense | 1 |  |
|  | growth | 3 |  |
|  | employment | 1 |  |
|  | private | 1 |  |
|  | expense | 1 |  |
|  | business | 5 |  |
| Inequality | status | 3 | 27 |
|  | employment | 2 |  |
|  | homeless | 2 |  |
|  | income | 15 |  |
|  | social inequality | 1 |  |
|  | unaffordability | 1 |  |
|  | overcrowded | 1 |  |
|  | job | 1 |  |
|  | affordable housing | 2 |  |
|  | poverty | 7 |  |
|  | housing | 9 |  |
|  | amenities | 2 |  |
|  | poorer quality building | 1 |  |
|  | precarious housing | 1 |  |
|  | poor quality services | 1 |  |
|  | outdoor worker | 1 |  |
|  | tourists | 1 |  |
|  | middle class | 1 |  |
|  | wealth | 1 |  |

Note: After reading and summarizing each adaptation plan, we identified the economic factors, and subdivided them into related terms. Within each of these, we developed mention counters to capture the emphases of the narrative. While “mentions” count the number of times each plan mentions a term, in "total", if two or more terms are mentioned in the same plan, they only count as one. This removes the problem of double counting.

**Table 2C: Techno-infrastructural Factors Receiving Attention in the Adaptation Plans**

| **Factor** | **Term** | **Mentions** | **Total** |  | **Factor** | **Term** | **Mentions** | **Total** |
| --- | --- | --- | --- | --- | --- | --- | --- | --- |
| Water Systems | water | 19 | 20 |  | Built-environment | building | 16 | 32 |
|  | sewage | 0 |  |  |  | housing | 7 |  |
|  | hydropower | 1 |  |  |  | impermeable | 4 |  |
|  | water supply | 5 |  |  |  | slum | 0 |  |
|  | groundwater | 1 |  |  |  | housing | 7 |  |
|  | sewer | 8 |  |  |  | house | 3 |  |
|  | waste | 1 |  |  |  | property | 3 |  |
|  | dyke | 1 |  |  |  | infrastructure | 25 |  |
|  | sanitation | 3 |  |  |  | facilities | 5 |  |
| Energy Systems | energy system | 1 | 14 |  |  | properties | 2 |  |
|  | hydropower | 1 |  |  |  | urban design | 0 |  |
|  | energy | 9 |  |  |  | UHI | 0 |  |
|  | electricity | 6 |  |  |  | urban heat island | 9 |  |
|  | thermal power | 1 |  |  |  | impervious | 1 |  |
|  | power | 9 |  |  | Inequality | unaffordability | 1 | 24 |
| Food Systems | agricultural | 1 | 6 |  |  | overcrowded | 1 |  |
|  | food access | 4 |  |  |  | affordable housing | 2 |  |
|  | food | 1 |  |  |  | housing | 9 |  |
|  | crops | 1 |  |  |  | poorer quality building | 1 |  |
| Transportation | transportation | 5 | 10 |  |  | precarious housing | 1 |  |
|  | road | 3 |  |  |  | poor quality services | 1 |  |
|  | tunnel | 1 |  |  |  | infrastructure | 3 |  |
|  | bridge | 0 |  |  |  | amenities | 2 |  |
|  | transit | 2 |  |  |  |  |  |  |
|  | traffic | 1 |  |  |  |  |  |  |

Note: After reading and summarizing each adaptation plan, we identified the infrastructural factors, and subdivided them into related terms. Within each of these, we developed mention counters to capture the emphases of the narrative. While “mentions” count the number of times each plan mentions a term, in "total", if two or more terms are mentioned in the same plan, they only count as one. This removes the problem of double counting.

**Table 2D: Environmental Factors Receiving Attention in the Adaptation Plans**

| Environmental | | | |
| --- | --- | --- | --- |
| Factor | Term | Mentions | Total |
| Geographic | coast | 8 | 16 |
|  | river | 5 |  |
|  | location | 8 |  |
|  | geography | 1 |  |
|  | geographic | 0 |  |
|  | shoreline | 0 |  |
| Topographic | mountain | 5 | 16 |
|  | landslide | 5 |  |
|  | topography | 6 |  |
|  | low-lying | 4 |  |
|  | low lying | 0 |  |
|  | subsiding | 1 |  |
|  | ground level | 0 |  |
| Others | natural resource | 0 | 6 |
|  | habitat | 0 |  |
|  | green | 5 |  |
|  | park | 1 |  |
|  | environmental service | 4 |  |
|  | wildlife | 0 |  |
|  | grass | 0 |  |

Note: After reading and summarizing each adaptation plan, we identified the environmental factors, and subdivided them into related terms. Within each of these, we developed mention counters to capture the emphases of the narrative. While “mentions” count the number of times each plan mentions a term, in "total", if two or more terms are mentioned in the same plan, they only count as one. This removes the problem of double counting.

**Table 2E: Governance Factors Receiving Attention in the Adaptation Plans**

| Governance | | | |
| --- | --- | --- | --- |
| Factor | Term | Mentions | Total |
| Governance | jurisdictional coordination | 1 | 20 |
|  | planning | 5 |  |
|  | fragmentation | 1 |  |
|  | monitoring | 1 |  |
|  | policies | 1 |  |
|  | management | 3 |  |
|  | law | 1 |  |
|  | education | 1 |  |
|  | emergency | 2 |  |
|  | regulation | 2 |  |
|  | standard | 1 |  |
|  | policy | 2 |  |
| Inequality | informal | 7 | 14 |
|  | power | 1 |  |
|  | information | 2 |  |
|  | healthcare | 1 |  |
|  | cooling center | 1 |  |
|  | marginalization | 1 |  |
|  | health inequalities | 1 |  |

Note: After reading and summarizing each adaptation plan, we identified governance factors, and subdivided them into related terms. Within each of these, we developed mention counters to capture the emphases of the narrative. While “mentions” count the number of times each plan mentions a term, in "total", if two or more terms are mentioned in the same plan, they only count as one. This removes the problem of double counting.

**Table 3A: Techno-infrastructural Actions Receiving Attention in the Adaptation Plans**

| Technical & Infrastructural | | | |
| --- | --- | --- | --- |
| Factor | Term | Mentions | Total |
| Infrastructure in General | coastal | 3 | 50 |
|  | infrastructure | 28 |  |
|  | recycling | 5 |  |
|  | maintenance | 2 |  |
|  | treatment | 3 |  |
|  | resiliency | 1 |  |
|  | efficiency | 10 |  |
|  | building | 22 |  |
|  | technology | 1 |  |
|  | waste | 12 |  |
|  | utilities | 1 |  |
| Energy | grid | 2 | 23 |
|  | electricity | 6 |  |
|  | renewable | 11 |  |
|  | solar | 4 |  |
|  | wind | 3 |  |
|  | energy | 19 |  |
| Water | water | 31 | 31 |
|  | water treatment | 1 |  |
|  | Storm-water | 7 |  |
|  | pumping | 1 |  |
|  | sanitation | 3 |  |
|  | rainwater | 5 |  |
| Food | food | 3 | 3 |
|  | agriculture | 1 |  |
| Transport | streets | 3 | 17 |
|  | transit | 7 |  |
|  | streets | 3 |  |
|  | car | 6 |  |
|  | transportation | 5 |  |
|  | technology | 1 |  |

Note: After reading and summarizing each adaptation plan, we identified techno-infrastructural actions and related terms. Within each of these, we developed mention counters, based on mention of the terms, to capture the emphases of the narratives.

**Table 3B- Institutional-behavioral Actions Receiving Attention in the Adaptation Plans**

| Institutional-behavioral | | | |
| --- | --- | --- | --- |
| Factor | Term | Mentions | Total |
| Awareness &  Knowledge | understanding | 5 | 31 |
|  | educate | 5 |  |
|  | information | 9 |  |
|  | train | 4 |  |
|  | awareness | 14 |  |
|  | aware | 14 |  |
|  | knowledge | 6 |  |
|  | outreach | 3 |  |
|  | capacity | 10 |  |
|  | communicate | 2 |  |
|  | education | 9 |  |
|  | research | 3 |  |
| Monitoring | monitor | 11 | 29 |
|  | anticipate | 1 |  |
|  | assess | 11 |  |
|  | evaluate | 1 |  |
|  | map | 7 |  |
|  | plan | 21 |  |
|  | planning | 1 |  |
|  | information gathering | 1 |  |
| Institution building | collaboration | 3 | 14 |
|  | council | 2 |  |
|  | policy | 4 |  |
|  | office | 3 |  |
|  | collaborate | 1 |  |
|  | leader | 2 |  |
|  | organization | 4 |  |
| Emergency  response | disaster | 8 | 22 |
|  | emergency response | 7 |  |
|  | emergency preparedness | 1 |  |
|  | warning | 12 |  |
| Urban planning | green space | 1 | 22 |
|  | urban planning | 1 |  |
|  | sea level rise | 2 |  |
|  | planning law | 1 |  |
|  | resilience | 6 |  |
|  | urban | 10 |  |
|  | zoning | 6 |  |
|  | ordinance | 2 |  |
|  | residential | 2 |  |
|  | neighborhood | 3 |  |

Note: After reading and summarizing each adaptation plan, we identified institutional-behavioral actions and related terms. Within each of these, we developed mention counters, based on mention of the terms, to capture the emphases of the narratives

**Table 3C: Economic Actions Receiving Attention in the Adaptation Plans**

| Economic | | | |
| --- | --- | --- | --- |
| Factor | Term | Mentions | Total |
| Instruments | measure | 4 | 24 |
|  | tourism | 2 |  |
|  | program | 10 |  |
|  | incentivize | 1 |  |
|  | incentive | 3 |  |
|  | code | 6 |  |
|  | economy | 4 |  |
|  | jobs | 6 |  |
| Financing | fund | 9 | 14 |
|  | finance | 3 |  |
|  | loan | 2 |  |
|  | grant | 0 |  |
|  | financial | 3 |  |
|  | invest | 8 |  |
|  | financing | 2 |  |

Note: After reading and summarizing each adaptation plan, we identified economic actions and related terms. Within each of these, we developed mention counters, based on mention of the terms, to capture the emphases of the narratives.

**Table 3D: Environmental Actions Receiving Attention in the Adaptation Plans**

| Environment and Conservation | | | |
| --- | --- | --- | --- |
| Factor | Term | Mentions | Total |
| Environment | biodiversity | 10 | 20 |
|  | ecosystems | 5 |  |
|  | green spaces | 4 |  |
|  | coastal | 1 |  |
|  | rainwater | 2 |  |
|  | parks | 3 |  |
|  | open spaces | 1 |  |
|  | flora | 2 |  |
|  | fauna | 2 |  |
|  | conservation | 5 |  |
|  | environment | 3 |  |

Note: After reading and summarizing each adaptation plan, we identified environmental actions and related terms. Within each of these, we developed mention counters, based on mention of the terms, to capture the emphases of the narratives.
